# Supplementary material for: Structural Characterization, Rheology, Texture, and Potential Hypoglycemic Effect of Polysaccharides from Brasenia schreberi
Source: Foods. 2025 May 21;14(10):1836. doi: 10.3390/foods14101836 (PMC12111196; doi:10.3390/foods14101836)
Supplement: Supplementary file 1 [file foods-14-01836-s001.zip › foods-3574898-supplementary.pdf]

Structural Characterization, Rheology, Texture, and Potential Hypoglycemic Effect of Polysaccharides from *Brasenia schreberi*

Zhangli Jia, Yin Chen, Chunyu Niu, Yan Xu * and Yan Chen *

College of Food and Pharmacy, Zhejiang Ocean University, 1 South Haida Road, Zhoushan 316000, China; jiazhangli@zjou.edu.cn (Z.J.); mojojo1984@163.com (Y.C.); niuchunyu0808@126.com (C.N.)

* Correspondence: xuyan@zjou.edu.cn (Y.X.); 2022067@zjou.edu.cn (Y.C.)

Supplementary materials:

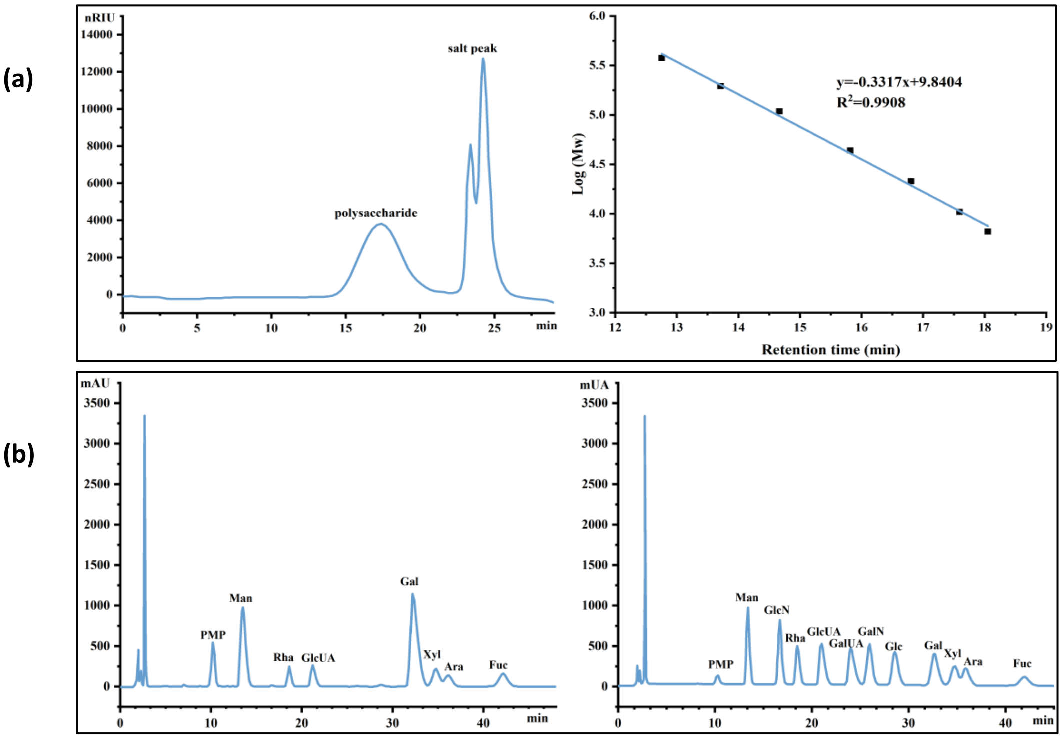

Figure S1. HPGPC and HPLC chromatogram of BSP. (a) HPGPC chromatogram of BSP and the standard curve of molecular weights. (b) HPLC chromatogram of BSP and standard monosaccharide.
